# Association between γ-Glutamyl Transferase and Metabolic Syndrome: A Cross-Sectional Study of an Adult Population in Beijing

**DOI:** 10.3390/ijerph10115523

**Published:** 2013-10-29

**Authors:** Lixin Tao, Xia Li, Huiping Zhu, Yue Gao, Yanxia Luo, Wei Wang, Zhaoping Wang, Dongning Chen, Lijuan Wu, Xiuhua Guo

**Affiliations:** 1 School of Public Health, Capital Medical University, 10 Xitoutiao, Youanmen, Beijing 100069, China; E-Mails: taolixin.2008@163.com (L.T.); lixia_new@163.com (X.L.); zhuhuiping79@163.com (H.Z.); lyx100@ccmu.edu.cn (Y.L.); wujuan811017@163.com (L.W.); 2 Beijing Municipal Key Laboratory of Clinical Epidemiology, 10 Xitoutiao, Youanmen, Beijing 100069, China; 3 Department of Epidemiology & Public Health, University College Cork, Western Road, Cork 78746, Ireland; 4 Beijing Anzhen Hospital, Capital Medical University, 2 Anzhen Road, Chaoyang, Beijing 100029, China; E-Mail: yp7391@sina.com; 5 School of Medical Science, Edith Cowan University, 2 Bradford Street, Mount Lawley, WA 6050, Australia; E-Mail: wei6014@yahoo.com; 6 Physical Examination Department, Beijing Tongren Hospital, Capital Medical University, 1 Dongjiaominxiang, Beijing 100041, China; E-Mails: doctorzpw@yahoo.com.cn (Z.W.); chendn@trhos.com (D.C.)

**Keywords:** liver enzymes, metabolic syndrome, gamma-glutamyl transferase, diagnosis

## Abstract

The relationship between liver enzymes and clustered components of metabolic syndrome (MetS) is explored and the predictive power of γ-glutamyl transferase (GGT) for the diagnosis of MetS in an adult population in Beijing is investigated. A total of 10,553 adults aged 20–65 years who underwent health examinations at Beijing Tongren Hospital in 2012 were enrolled in the study. Multivariate logistic regression analysis is conducted to determine the associations between the levels of various liver enzymes and clustered components of MetS. A receiver operating characteristic analysis is used to determine the optimal cut-off value of GGT for the diagnosis of MetS. A high level of GGT is found to be positively associated with clustered components of MetS in both men and women after adjusting for age, body mass index (BMI), history of alcoholic fatty liver, and the presence of taking anti-hypertensive, anti-dyslipidemic, and anti-diabetic drugs. Among all components of MetS, GGT is more predictive of triglyceride, and BMI. The area-under-the-curve values of GGT for discriminating MetS from normal metabolic status in men and women are 0.73 and 0.80, respectively. The optimal cut-off value of GGT for men is 31.50 U/L, demonstrating a sensitivity of 74.00% and specificity of 62.00%. For women, it is 19.50 U/L (sensitivity 76.00% and specificity 70.00%). GGT is therefore recommended as a useful diagnostic marker for MetS, because the test is inexpensive, highly sensitive, and frequently encountered in clinical practice.

## 1. Introduction

γ-Glutamyl transferase (GGT) is independently associated with several pathological conditions, including cardiovascular disease (CVD) [1,2], diabetes [3,4,5], and metabolic syndrome (MetS) [6,7,8,9]. The enzyme is involved in glutathione metabolism and plays critical roles in antioxidant defense, detoxification, and inflammation processes. Moreover, it has recently been found to be involved in many physiological disorders, such as Parkinson’s disease and diabetes [10]. Also oxidative stress is suggested to be involved in the onset of several obesity-related disorders such as hypertension, dyslipidemia, type-2 diabetes mellitus and MetS [11]. MetS is a cluster of risk factors that include abdominal obesity, hyperglycaemia, raised blood pressure (BP), low high-density lipoprotein (HDL-C) and high triglyceride (TG). The prevalence of MetS is growing rapidly and is associated with an increased risk of nonalcoholic fatty liver disease, diabetes, CVD, and total mortality [12,13], thus the identification of biomarkers for MetS is of pivotal importance. 

Based on the available biochemical and clinical evidence, an association between high levels of GGT and MetS has been hypothesized. Many large-scale prospective studies have reported that high levels of GGT, even when within the normal range [14,15,16,17], is a strong and independent predictor of increased risk of stroke, cardiovascular mortality, and a number of MetS components [4,18,19,20,21]. These findings suggest the possibility that a high level of GGT might reflect the development of MetS and is, in general, independent of the effects of other metabolic risk factors. Other liver enzymes, including alanine aminotransferase (ALT), aspartate aminotransferase (AST), and alkaline phosphatase (ALP) were also reported to be positively related to an increased risk of MetS and related disorders [22,23,24,25,26]. Many references have suggested that liver enzymes are emerging as biomarkers of MetS and its clustering components in adolescents. However, it is not fully understood which liver enzyme are better indicators of MetS or clustering of its components.

Several studies have investigated the association between GGT and MetS, as well as the optimal cut-off values for this biomarker [26,27]. Other studies have explored the association between GGT and MetS in Chinese populations in particular [21,28]. However, inferences about the optimal cut-off values of GGT for the diagnosis of MetS have not been reported. The present study was designed to explore the relationship between GGT and clustered components of MetS. Moreover, it was designed to determine the optimal cut-off values for GGT that could be used to discriminate MetS from normal metabolic conditions.

## 2. Methods

### 2.1.Study Population

This cross-sectional study was designed to enroll a population of adults from Beijing who attended health examinations in 2012 at Beijing Tongren Hospital. In total, 13,613 subjects were enrolled. We excluded 3,060 patients with a history of CVD, myocardial infarction, cerebral infarction, gastric cancer, coronary artery bypass surgery, coronary stenting surgery, gastrectomy, and/or liver cirrhosis. Thus, a total of 10,553 adults living in Beijing (4,764 men and 5,789 women) were included in the final analysis. This study was approved by the ethics committee of Capital Medical University in Beijing (approval number: 2013SY26). All participants provided informed written consent.

### 2.2. Measurements

Information about medication use was gathered by trained medical staff during a standardized interview. Subjects who reported taking anti-hypertensive, anti-dyslipidemic or anti-diabetic drugs were considered to have elevated BP, elevated TG, reduced HDL-C or elevated fasting plasma glucose (FPG).

The participants underwent routine physical examinations that included the measurement of height, weight, BP, and overnight fasting blood sampling. Weight and height were measured without shoes, and body mass index (BMI) was calculated as weight (kg) divided by squared height (m). BP was measured on the right arm of subjects seated and at rest for at least 5-min by a trained nurse. During the 30 min preceding the measurements, the subjects were required to refrain from smoking or consuming caffeine. Three systolic and diastolic blood pressures were recorded, with an interval of at least 1 min between readings, and the average of the last two measurements were used for data analysis.

Blood samples were obtained from antecubital vein in the morning after an overnight fasting period and placed in tubes containing EDTA. HDL-C, TG, FPG, GGT, ALT, AST, and ALP were measured enzymatically using a chemistry analyzer (Beckman LX 20, Pasadena, CA, USA) at the central laboratory of the hospital. Serum GGT was assayed using the standard method recommended by the International Federation for Clinical Chemistry. That is, L-γ-glutamyl-3-carboxy-4-nitroanilide was used as substrate at a temperature of 37 °C with a normal reference range of 11–50 U/L for men, and 7–32 U/L for women [29]. The normal laboratory reference ranges for ALT, AST, and ALP are 0–40, 0–40, and 15–112 U/L, respectively. The sensitivity of the assay is 2 U/L. All analyses were performed in accordance with the manufacturer’s recommendations. The intra and inter-assay coefficients of variation for all laboratory tests were under 5%.

### 2.3. Definition of MetS

MetS was diagnosed if the subjects had three or more risk determinants according to the Joint Interim Statement criteria [30]. However, in this study, waist circumference (WC) was not measured because of limited health check-up site, and BMI was taken as a substitute for the component of obesity [31]. The determinants were as follows:

1. Obesity: BMI ≥ 28 kg/m²;
2. Elevated TG (drug treatment for elevated TG is an alternate indicator) ≥ 150 mg/dL (1.7 mmol/L);
3. Reduced HDL-C (drug treatment for reduced HDL-C is an alternate indicator) < 40 mg/dL (1.0 mmol/L) in males, <50 mg/dL (1.3 mmol/L) in females;
4. Elevated BP (anti-hypertensive drug treatment in a patient with a history of hypertension is an alternate indicator) systolic ≥ 130 mm Hg and/or diastolic ≥ 85 mm Hg; and,
5. Elevated FPG (drug treatment of elevated glucose is an alternate indicator) ≥ 100 mg/dL.

### 2.4. Data Analysis

Data was analyzed using the SAS software (version 9.2, SAS Institute, Cary, NC, USA), and *p* < 0.05 was considered as significant. To compare the differences between groups, Student’s *t* test or Wilcoxon rank sum test was used for continuous variables, and *χ*^2^ test or Fisher’s exact test was used for categorical variables. The odds ratios (ORs) and 95% confidence intervals (CIs) were assessed using multivariate logistic regression analysis to determine the associations between four liver enzymes and clustered components of MetS after adjusting for age, BMI, alcoholic fatty liver history, and the presence of prescriptive drug taking (anti-hypertensive, anti-dyslipidemic, and anti-diabetic drugs). The four liver enzymes levels were respectively classified into four groups using the 25th, 50th, and 75th percentiles as cut-points. Receiver operating characteristic (ROC) curve was used to determine the optimal cut-off values for these four liver enzymes in terms of their sensitivity and specificity for diagnosing MetS. The area under the curve (AUC) was calculated and 95% CI was estimated. 

## 3. Results

### 3.1. Prevalence of MetS and its Components

**Figure 1:**
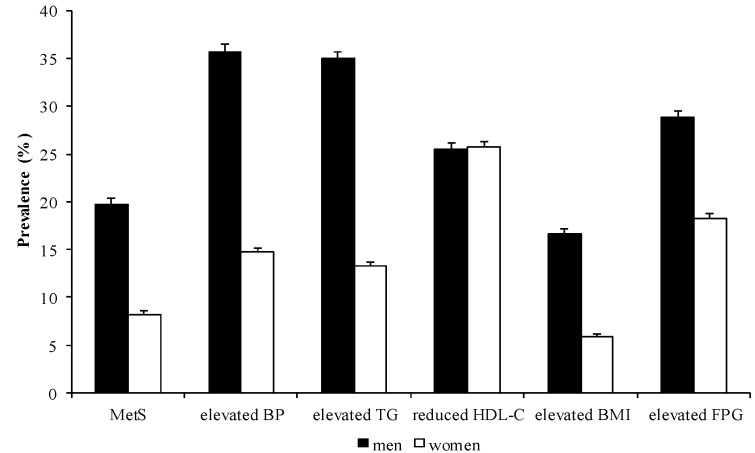
Prevalence of MetS and its components by sex.

Abbreviations: MetS = metabolic syndrome; BP = blood pressure; TG = triglyceride; HDL-C = high-density lipoprotein cholesterol; BMI = body mass index; FPG = fasting plasma glucose.

The sex-specific prevalence of MetS and its components are described in Table 1 and Figure 1. Overall, the prevalence of MetS among all subjects was 13.43%, with 19.75% of men and 8.22% of women diagnosed with MetS. It was shown that men have a higher prevalence of MetS, elevated BP, elevated TG, elevated BMI, and elevated FPG levels, whereas women demonstrated a slightly higher prevalence of reduced HDL-C.

### 3.2. Basic Characteristics and Hematological Parameters

The basic characteristics and hematological parameters of all subjects are shown in Table 2. In addition to the levels of five MetS components and four liver enzymes, the prevalence of history of alcoholic fatty liver, and taking anti-hypertensive, anti-dyslipidemic, and anti-diabetic drugs were significantly higher in the MetS group than the non-MetS group for both men and women.

**Table 1:** Prevalence of MetS and its components by gender.

Sex	Total	MetS		Elevated BP		Elevated TG		Reduced HDL-C		Elevated BMI		Elevated FPG	
		n	%	n	%	n	%	n	%	n	%	n	%
**Men**	4,764	941	19.75	1,702	35.73	1,666	34.97	1,214	25.48	794	16.67	1,371	28.78
**Women**	5,789	476	8.22	849	14.67	769	13.28	1,488	25.70	342	5.91	1,059	18.29
**Total**	10,553	1,417	13.43	2,551	24.17	2,435	23.07	2,702	25.60	1,136	10.76	2,430	23.03

n: The number of cases with MetS, or abnormal components; %: The prevalence of cases with MetS or abnormal components among male, female, or total subjects. Abbreviations: MetS = metabolic syndrome; BP = blood pressure; TG = triglyceride; HDL-C = high-density lipoprotein cholesterol; BMI = body mass index; FPG = fasting plasma glucose.

**Table 2:** Basic characteristics and hematological parameters by gender.

Variables	Men		*p* Value	Women		*p* Value
	MetS	non-MetS		MetS	non-MetS	
N	941	3,823	-	476	5,313	-
Age (year)	44 (35–53)	38 (31–49)	<0.0001 *^a^*	50 (43–57)	40 (30–49)	<0.0001 *^a^*
SBP (mmHg)	130 ± 15	118 ± 14	<0.0001 *^b^*	128 ± 17	111 ± 13	<0.0001 *^b^*
DBP (mmHg)	84 ± 10	75 ± 10	<0.0001 *^b^*	81 ± 10	70 ± 9	<0.0001 *^b^*
HDL-C (mmol/L)	0.97 ± 0.22	1.26 ± 0.29	<0.0001 *^b^*	1.13 ± 0.21	1.58 ± 0.34	<0.0001 *^b^*
TG (mmol/L)	2.97 ± 2.21	1.46 ± 1.18	<0.0001 *^b^*	2.34 ± 1.26	0.96 ± 0.60	<0.0001 *^b^*
FPG (mmol/L)	6.34 ± 1.79	5.36 ± 1.04	<0.0001 *^b^*	6.40 ± 1.81	5.17 ± 0.74	<0.0001 *^b^*
BMI (kg/m^2^)	28.21 ± 3.25	24.39 ± 4.02	<0.0001 *^b^*	27.41 ± 3.71	22.05 ± 2.93	<0.0001 *^b^*
GGT (U/L)	52.09 ± 31.74	34.01 ± 22.97	<0.0001 *^b^*	33.53 ± 23.17	19.92 ± 13.51	<0.0001 *^b^*
ALT (U/L)	45.58 ± 32.17	30.95 ± 30.22	<0.0001 *^b^*	31.84 ± 19.28	20.38 ± 19.59	<0.0001 *^b^*
AST (U/L)	37.50 ± 14.52	31.05 ± 19.26	<0.0001 *^b^*	34.64 ± 13.25	27.54 ± 11.98	<0.0001 *^b^*
ALP (U/L)	65.68 ± 15.87	62.35 ± 15.10	<0.0001 *^b^*	65.18 ± 18.31	53.43 ± 16.19	<0.0001 *^b^*
History of alcoholic fatty liver, n (%)	27 (2.87)	41 (1.07)	<0.0001 *^c^*	8 (1.68)	11 (0.21)	<0.0001 *^c^*
Anti-hypertensive drugs, n (%)	256 (27.21)	346 (9.05)	<0.0001 *^c^*	174 (36.55)	337 (6.34)	<0.0001 *^c^*
Anti-dyslipidemic drugs, n (%)	205 (21.79)	206 (5.39)	<0.0001 *^c^*	90 (18.91)	272 (5.12)	<0.0001 *^c^*
Anti-diabetic drugs, n (%)	80 (8.50)	93 (2.56)	<0.0001 *^c^*	49 (10.29)	92 (1.73)	<0.0001 *^c^*

Abbreviations: MetS = metabolic syndrome; SBP = systolic blood pressure; DBP = diastolic blood pressure; HDL-C = high-density lipoprotein cholesterol; TG = triglyceride; FPG = fasting plasma glucose; BMI = body mass index; GGT = gamma-glutamyl transferase; ALT = alanine aminotransferase; AST = aspartate aminotransferase; ALP = alkaline phosphatase. *^a^* Determined using the rank-sum test; *^b^* Determined using *t* test; *^c^* Determined using *χ*^2^ test.

### 3.3. Association between Liver Enzymes and MetS, as well as its Components

The associations between four liver enzymes and MetS, as well as its components, were explored using multivariate logistic regression model after adjusting for age, BMI, history of alcoholic fatty liver, and the presence of taking anti-hypertensive, anti-dyslipidemic, and anti-diabetic drugs.

The associations between the four liver enzymes and MetS are shown in Table 3. Compared with the first quartile group, the adjusted OR of GGT for indicating MetS increased from 1.40 (95% CI: 1.09–1.96) to 3.50 (95% CI: 2.50–4.91) for men and from 1.80 (95% CI: 1.04–3.10) to 5.61 (95% CI: 3.41–9.23) for women. ALT was significantly associated with MetS in quartile 3 and 4 for men, and in quartile 4 for women. AST was associated with MetS in quartile 4 for both men and women. ALP was associated with MetS in quartiles 3 and 4 for women, and in quartile 4 for men. 

**Table 3:** Factors associated with MetS by gender.

	Q1	Q2	Q3	Q4
***Men***				
GGT (U/L)	≤20	21–29	30–43	≥44
Cases/subjects at risk	64/1,178	151/1,213	261/1,167	465/1,206
OR (95% CI: lower–upper)	1.00	1.40 (1.09–1.96)	2.09 (1.50–2.91)	3.50 (2.50–4.91)
ALT (U/L)	≤19	20–27	28–38	≥39
Cases/subjects at risk	98/1,191	176/1,201	242/1,188	425/1,184
OR (95% CI: lower–upper)	1.00	1.23 (0.90–1.66)	1.43 (1.05–1.95)	1.81 (1.28–2.55)
AST (U/L)	≤24	25–30	31–36	≥37
Cases/subjects at risk	129/1,214	188/1,294	222/1,145	402/1,111
OR (95% CI: lower–upper)	1.00	1.09 (0.82­–1.45)	1.07 (0.80–1.43)	1.62 (1.19–2.20)
ALP (U/L)	≤52	53–61	62–71	≥72
Cases/subjects at risk	198/1,288	217/1,106	244/1,202	282/1,168
OR (95% CI: lower–upper)	1.00	1.24 (0.96–1.61)	1.29 (0.99–1.66)	1.48 (1.16–1.90)
***Women***				
GGT (U/L)	≤13	14–17	18–22	≥23
Cases/subjects at risk	22/1,843	44/1,386	100/1,151	310/1,409
OR (95% CI: lower–upper)	1.00	1.80 (1.04–3.10)	3.49 (2.11–5.78)	5.61 (3.41–9.23)
ALT (U/L)	≤13	14–18	19–23	≥24
Cases/subjects at risk	27/1,480	64/1,624	98/1,248	287/1,437
OR (95% CI: lower–upper)	1.00	1.30 (0.79–2.13)	1.44 (0.88–2.35)	1.95 (1.19–3.21)
AST (U/L)	≤22	23–26	27–31	≥32
Cases/subjects at risk	36/1,451	80/1,494	110/1,434	250/1,410
OR (95% CI: lower–upper)	1.00	1.62 (1.04–2.54)	1.33 (0.85–2.07)	2.01 (1.29–3.15)
ALP (U/L)	≤42	43–51	52–63	≥64
Cases/subjects at risk	43/1,556	66/1,352	141/1,519	226/1,362

Abbreviations: MetS = metabolic syndrome; GGT = gamma-glutamyl transferase; ALT = alanine aminotransferase; AST = aspartate aminotransferase; ALP = alkaline phosphatase; OR = odds ratio; CI = confidence interval.

The associations between the four liver enzymes and elevated BP are shown in Table 4. The adjusted OR of GGT for indicating elevated BP increased from 1.24 (95% CI: 1.02–1.51) to 2.18 (95% CI: 1.76–2.70) for men, while GGT was positively associated with elevated BP in quartile 3 for women. Risk of elevated BP increased with an increase in ALT level for men, and ALT was significantly associated with elevated BP in quartile 3 and 4 for women. No positive association was found between AST and elevated BP for men and women. ALP was positively associated with elevated BP in quartile 3 and 4 for women, but no association was found for men.

**Table 4:** Factors associated with elevated BP by gender.

	Q1	Q2	Q3	Q4
***Men***				
GGT (U/L)	≤20	21–29	30–43	≥44
n (%)	272/1,178	374/1,213	453/1,167	603/1,206
OR (95% CI: lower–upper)	1.00	1.24 (1.02–1.51)	1.48 (1.21–1.81)	2.18 (1.76–2.70)
ALT (U/L)	≤19	20–27	28–38	≥39
n (%)	304/1,191	410/1,201	464/1,188	524/1,184
OR (95% CI: lower–upper)	1.00	1.26 (1.05–1.53)	1.41 (1.16–1.72)	1.45 (1.16–1.80)
AST (U/L)	≤24	25–30	31–36	≥37
n (%)	340/1,214	463/1,294	408/1,145	491/1,111
OR (95% CI: lower–upper)	1.00	1.20 (0.99–1.44)	0.97 (0.80–1.18)	1.04 (0.83–1.30)
ALP (U/L)	≤52	53–61	62–71	≥72
n (%)	437/1,288	381/1,106	402/1,202	482/1,168
OR (95% CI: lower–upper)	1.00	0.94 (0.78–1.12)	0.86 (0.72–1.03)	1.16 (0.97–1.38)
***Women***				
GGT (U/L)	≤13	14–17	18–22	≥23
Cases/subjects at risk	142/1,843	155/1,386	210/1,151	342/1,409
OR (95% CI: lower–upper)	1.00	1.15 (0.89–1.48)	1.46 (1.13–1.88)	1.28 (0.98–1.66)
ALT (U/L)	≤13	14–18	19–23	≥24
Cases/subjects at risk	111/1,480	187/1,624	202/1,248	349/1,437
OR (95% CI: lower–upper)	1.00	1.26 (0.97–1.63)	1.40 (1.07–1.83)	1.68 (1.27–2.21)
AST (U/L)	≤22	23–26	27–31	≥32
Cases/subjects at risk	134/1,451	172/1,494	233/1,434	310/1,410
OR (95% CI: lower–upper)	1.00	0.95 (0.73–1.23)	1.12 (0.86–1.44)	1.15 (0.87–1.52)
ALP (U/L)	≤42	43–51	52–63	≥64
Cases/subjects at risk	109/1,556	136/1,352	254/1,519	350/1,362
OR (95% CI: lower–upper)	1.00	1.26 (0.95–1.65)	1.79 (1.39–2.31)	1.87 (1.43–2.45)

Abbreviations: BP = blood pressure; GGT = gamma-glutamyl transferase; ALT = alanine aminotransferase; AST = aspartate aminotransferase; ALP = alkaline phosphatase; OR = odds ratio; CI = confidence interval.

The associations between the four liver enzymes and elevated TG are shown in Table 5. The adjusted OR of GGT for predicting elevated TG increased from 2.03 (95% CI: 1.63–2.55) to 4.79 (95% CI: 3.78–6.08) for men, and from 1.73 (95% CI: 1.22–2.47) to 5.15 (95% CI: 3.70–7.16) for women. Risk for elevated TG increased with the increase in ALT level for men, and ALT was significantly associated with elevated TG in quartile 3 and 4 for women. AST was significantly associated with elevated TG in quartile 3 and 4 for men, and in quartile 4 for women. Positive associations were found between ALP and elevated TG for men and women.

**Table 5:** Factors associated with elevated TG by gender.

	Q1	Q2	Q3	Q4
***Men***				
GGT (U/L)	≤20	21–29	30–43	≥44
Cases/subjects at risk	212/1,178	272/1,213	328/1,167	402/1,206
OR (95% CI: lower–upper)	1.00	2.03 (1.63–2.55)	3.10 (2.47–3.89)	4.79 (3.78–6.08)
ALT (U/L)	≤19	20–27	28–38	≥39
Cases/subjects at risk	160/1,191	283/1,201	297/1,188	444/1,184
OR (95% CI: lower–upper)	1.00	1.38 (1.12–1.71)	1.56 (1.26–1.94)	1.74 (1.36–2.23)
AST (U/L)	≤24	25–30	31–36	≥37
Cases/subjects at risk	272/1,214	264/1,294	298/1,145	380/1,111
OR (95% CI: lower–upper)	1.00	1.21 (0.99–1.47)	1.44 (1.17–1.77)	1.75 (1.39–2.20)
ALP (U/L)	≤52	53–61	62–71	≥72
Cases/subjects at risk	241/1,288	289/1,106	322/1,202	362/1,168
OR (95% CI: lower–upper)	1.00	1.29 (1.06–1.56)	1.31 (1.09–1.52)	1.40 (1.16–1.69)
***Women***				
GGT (U/L)	≤13	14–17	18–22	≥23
Cases/subjects at risk	302/1,843	299/1,386	334/1,151	553/1,409
OR (95% CI: lower–upper)	1.00	1.73 (1.22–2.47)	3.68 (2.65–5.11)	5.15 (3.70–7.16)
ALT (U/L)	≤13	14–18	19–23	≥24
Cases/subjects at risk	280/1,480	358/1,624	334/1,248	516/1,437
OR (95% CI: lower–upper)	1.00	1.39 (0.99–1.94)	1.62 (1.15–2.27)	1.81 (1.27–2.57)
AST (U/L)	≤22	23–26	27–31	≥32
Cases/subjects at risk	307/1,451	350/1,494	361/1,434	470/1,410
OR (95% CI: lower–upper)	1.00	1.24 (0.91–1.69)	1.22 (0.90–1.65)	1.68 (1.24–2.30)
ALP (U/L)	≤42	43–51	52–63	≥64
Cases/subjects at risk	271/1,556	331/1,352	428/1,519	458/1,362
OR (95% CI: lower–upper)	1.00	1.45 (1.06–1.99)	1.72 (1.29–2.31)	2.06 (1.53–2.78)

Abbreviations: TG = triglyceride; GGT = gamma-glutamyl transferase; ALT = alanine aminotransferase; AST = aspartate aminotransferase; ALP = alkaline phosphatase; OR = odds ratio; CI = confidence interval.

The associations between the four liver enzymes and reduced HDL-C are shown in Table 6. The adjusted OR of GGT for predicting reduced HDL-C increased from 1.30 (95% CI: 1.08–1.55) to 2.38 (95% CI: 1.98–2.85) for women, but no positive association was found for men. ALT was positively associated with reduced HDL-C for men, but no association was shown for women. The association between AST and reduced HDL-C was not found for men and women. ALP was significantly associated with reduced HDL-C for both men and women.

**Table 6:** Factors associated with reduced HDL-C by gender.

	Q1	Q2	Q3	Q4
***Men***				
GGT (U/L)	≤20	21–29	30–43	≥44
Cases/subjects at risk	212/1,178	272/1,213	328/1,167	402/1,206
OR (95% CI: lower–upper)	1.00	1.04 (0.84–1.28)	1.14 (0.92–1.43)	1.20 (0.95–1.51)
ALT (U/L)	≤19	20–27	28–38	≥39
Cases/subjects at risk	191/1,191	283/1,201	297/1,188	444/1,184
OR (95% CI: lower–upper)	1.00	1.54 (1.25–1.91)	1.61 (1.29–2.00)	2.45 (1.92–3.11)
AST (U/L)	≤24	25–30	31–36	≥37
Cases/subjects at risk	272/1,214	264/1,294	298/1,145	380/1,111
OR (95% CI: lower–upper)	1.00	0.73 (0.60­–0.89)	0.81 (0.66–1.00)	0.84 (0.66–1.06)
ALP (U/L)	≤52	53–61	62–71	≥72
Cases/subjects at risk	241/1,288	289/1,106	322/1,202	362/1,168
OR (95% CI: lower–upper)	1.00	1.50 (1.23–1.84)	1.53 (1.26–1.86)	1.78 (1.46–2.16)
***Women***				
GGT (U/L)	≤13	14–17	18–22	≥23
Cases/subjects at risk	302/1,843	299/1,386	334/1,151	553/1,409
OR (95% CI: lower–upper)	1.00	1.30 (1.08–1.55)	1.78 (1.48–2.14)	2.38 (1.98–2.85)
ALT (U/L)	≤13	14–18	19–23	≥24
Cases/subjects at risk	280/1,480	358/1,624	334/1,248	516/1,437
OR (95% CI: lower–upper)	1.00	1.05 (0.87–1.26)	1.13 (0.92–1.38)	1.22 (0.98–1.53)
AST (U/L)	≤22	23–26	27–31	≥32
Cases/subjects at risk	307/1,451	350/1,494	361/1,434	470/1,410
OR (95% CI: lower–upper)	1.00	0.99 (0.83–1.20)	0.90 (0.74–1.09)	1.01 (0.82–1.24)
ALP (U/L)	≤42	43–51	52–63	≥64
Cases/subjects at risk	271/1,556	331/1,352	428/1,519	458/1,362
OR (95% CI: lower–upper)	1.00	1.39 (1.15–1.67)	1.53 (1.28–1.83)	1.68 (1.38–2.05)

Abbreviations: HDL-C = high-density lipoprotein cholesterol; GGT = gamma-glutamyl transferase; ALT = alanine aminotransferase; AST = aspartate aminotransferase; ALP = alkaline phosphatase; OR = odds ratio; CI = confidence interval.

The associations between the four liver enzymes and elevated FPG are shown in Table 7. The adjusted OR of GGT increased from 1.46 (95% CI: 1.16–1.84) to 2.38 (95% CI: 1.90–2.99) for women, and positive association between GGT and elevated FPG were shown in quartile 3 and 4 for men. ALT was significantly associated with elevated FPG in quartile 3 and 4 for women, but significant association was not found for women. AST was positively correlated with elevated FPG in quartile 4 for men, and in quartile 2 and 4 for women. ALP was significantly correlated with elevated FPG in quartile 3 and 4 for women, but no positive association was found for men.

**Table 7:** Factors associated with elevated FPG by gender.

	Q1	Q2	Q3	Q4
***Men***				
GGT (U/L)	≤20	21–29	30–43	≥44
Cases/subjects at risk	210/1,178	280/1,213	393/1,167	488/1,206
OR (95% CI: lower–upper)	1.00	1.15 (0.92–1.44)	1.75 (1.41–2.19)	2.23 (1.77–2.80)
ALT (U/L)	≤19	20–27	28–38	≥39
Cases/subjects at risk	275/1,191	346/1,201	345/1,188	405/1,184
OR (95% CI: lower–upper)	1.00	1.04 (0.84–1.29)	0.95 (0.75–1.19)	1.10 (0.84–1.44)
AST (U/L)	≤24	25–30	31–36	≥37
Cases/subjects at risk	277/1,214	329/1,294	330/1,145	435/1,111
OR (95% CI: lower–upper)	1.00	1.08 (0.88­–1.33)	1.13 (0.91–1.40)	1.56 (1.25–1.95)
ALP (U/L)	≤52	53–61	62–71	≥72
Cases/subjects at risk	356/1,288	314/1,106	332/1,202	369/1,168
OR (95% CI: lower–upper)	1.00	0.95 (0.77–1.16)	0.92 (0.75–1.12)	0.95 (0.78–1.15)
***Women***				
GGT (U/L)	≤13	14–17	18–22	≥23
Cases/subjects at risk	160/1,843	193/1,386	243/1,151	463/1,409
OR (95% CI: lower–upper)	1.00	1.46 (1.16–1.84)	1.90 (1.51–2.39)	2.38 (1.90–2.99)
ALT (U/L)	≤13	14–18	19–23	≥24
Cases/subjects at risk	147/1,480	210/1,624	251/1,248	451/1,437
OR (95% CI: lower–upper)	1.00	1.24 (0.95–1.62)	1.35 (1.02–1.79)	1.55 (1.15–2.09)
AST (U/L)	≤22	23–26	27–31	≥32
Cases/subjects at risk	154/1,451	231/1,494	250/1,434	424/1,410
OR (95% CI: lower–upper)	1.00	1.32 (1.05–1.68)	1.17 (0.93–1.49)	1.86 (1.48–2.35)
ALP (U/L)	≤42	43–51	52–63	≥64
Cases/subjects at risk	159/1,556	181/1,352	316/1,519	403/1,362
OR (95% CI: lower–upper)	1.00	1.25 (0.95–1.65)	1.78 (1.38–2.30)	1.85 (1.41–2.42)

The associations between the four liver enzymes and elevated BMI are shown in Table 8. The adjusted OR of GGT for indicating elevated BMI increased from 2.65 (95% CI: 1.86–3.78) to 4.97 (95% CI: 3.47–7.11) for men, and from 2.15 (95% CI: 1.26–3.67) to 5.88 (95% CI: 3.56–9.70) for women. Risk for elevated BMI increased with the increase in ALT level for men, and ALT was significantly associated with elevated BMI in quartile 3 and 4 for women. No significant association was found between AST, ALP and elevated BMI for men and women. 

**Table 8:** Factors associated with elevated BMI by gender.

	Q1	Q2	Q3	Q4
***Men***				
GGT (U/L)	≤20	21–29	30–43	≥44
Cases/subjects at risk	45/1,178	151/1,213	228/1,167	370/1,206
OR (95% CI: lower–upper)	1.00	2.65 (1.86–3.78)	3.28 (2.30–4.68)	4.97 (3.47–7.11)
ALT (U/L)	≤19	20–27	28–38	≥39
Cases/subjects at risk	72/1,191	141/1,201	191/1,188	390/1,184
OR (95% CI: lower–upper)	1.00	1.49 (1.09–2.03)	1.68 (1.23–2.29)	3.17 (2.33–4.32)
AST (U/L)	≤24	25–30	31–36	≥37
Cases/subjects at risk	116/1,214	157/1,294	203/1,145	318/1,111
OR (95% CI: lower–upper)	1.00	0.86 (0.65­–1.13)	0.96 (0.73–1.28)	1.00 (0.74–1.35)
ALP (U/L)	≤52	53–61	62–71	≥72
Cases/subjects at risk	188/1,288	194/1,106	215/1,202	197/1,168
OR (95% CI: lower–upper)	1.00	1.20 (0.94–1.52)	1.11 (0.88–1.41)	0.85 (0.67–1.09)
***Women***				
GGT (U/L)	≤13	14–17	18–22	≥23
Cases/subjects at risk	21/1,843	42/1,386	71/1,151	208/1,409
OR (95% CI: lower–upper)	1.00	2.15 (1.26–3.67)	3.32 (1.99–5.55)	5.88 (3.56–9.70)
ALT (U/L)	≤13	14–18	19–23	≥24
Cases/subjects at risk	22/1,480	50/1,624	80/1,248	190/1,437
OR (95% CI: lower–upper)	1.00	1.54 (0.92–2.58)	2.26 (1.36–3.75)	2.87 (1.74–4.72)
AST (U/L)	≤22	23–26	27–31	≥32
Cases/subjects at risk	36/1,451	52/1,494	97/1,434	157/1,410
OR (95% CI: lower–upper)	1.00	0.94 (0.60–1.48)	1.28 (0.83–1.96)	1.26 (0.81–1.96)
ALP (U/L)	≤42	43–51	52–63	≥64
Cases/subjects at risk	36/1,556	58/1,352	96/1,519	152/1,362
OR (95% CI: lower–upper)	1.00	1.38 (0.89–2.15)	1.56 (0.83–2.37)	1.75 (0.95–2.68)

Abbreviations: BMI = body mass index; GGT = gamma-glutamyl transferase; ALT = alanine aminotransferase; AST = aspartate aminotransferase; ALP = alkaline phosphatase; OR = odds ratio; CI = confidence interval.

Among all MetS components, GGT is more predictive of elevated TG and BMI according to Table 4, Table 5, Table 6, Table 7 and Table 8.

### 3.4. ROC Analysis of Various Liver Enzymes for the Diagnosis of MetS

**Figure 2:**
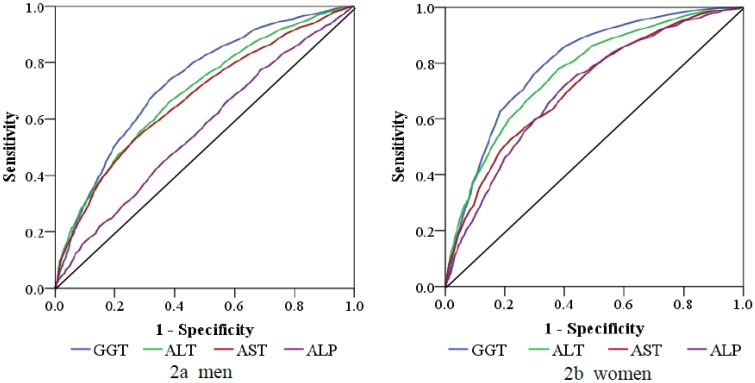
ROC analysis of GGT, ALT, AST, and ALP for the diagnosis of MetS.

Abbreviations: MetS = metabolic syndrome; GGT = gamma-glutamyl transferase; ALT = alanine aminotransferase; AST = aspartate aminotransferase; ALP = alkaline phosphatase; ROC = receiver operating characteristic; AUC = area under the curve.

The ROC analysis of GGT in comparison with the other liver enzymes is shown in Table 9 and Figure 2. The AUC was 0.73 (95% CI: 0.71–0.75) for GGT, 0.69 (95% CI: 0.67–0.71) for ALT, 0.67 (95% CI: 0.65–0.69) for AST, and 0.56 (95% CI: 0.54–0.58) for ALP for men. As for women, the AUC was 0.80 (95% CI: 0.78–0.82) for GGT, 0.76 (95% CI: 0.74–0.79) for ALT, 0.71 (95% CI: 0.69–0.74) for AST, and 0.70 (95% CI: 0.68–0.73) for ALP.

**Table 9:** ROC analysis of liver enzymes for the diagnosis of MetS.

	Variables	Area	95% CI	
			Lower	Upper
*****Men*****				
	GGT (U/L)	0.73	0.71	0.75
	ALT (U/L)	0.69	0.67	0.71
	AST (U/L)	0.67	0.65	0.69
	ALP (U/L)	0.56	0.54	0.58
*****Women*****				
	GGT (U/L)	0.80	0.78	0.82
	ALT (U/L)	0.76	0.74	0.79
	AST (U/L)	0.71	0.69	0.74
	ALP (U/L)	0.70	0.68	0.73

Abbreviations: MetS = metabolic syndrome; GGT = gamma-glutamyl transferase; ALT = alanine aminotransferase; AST = aspartate aminotransferase; ALP = alkaline phosphatase; ROC = receiver operating characteristics; CI = confidence interval.

The sensitivity, specificity, and GGT cut-off values for the diagnosis of MetS are presented in Table 10. According to the ROC curve analysis, the optimal cut-off value for GGT that can be used to diagnosis for MetS is 31.50 U/L, demonstrating a sensitivity of 74.00% and specificity of 62.00% for men, and the optimal cut-off value was 19.50 U/L with a sensitivity of 76.00% and specificity of 70.00% for women.

**Table 10:** Sensitivity and specificity values of specific GGT levels for the diagnosis of MetS.

	GGT (U/L)	Sensitivity	Specificity
***Men***			
	29.50	0.77	0.57
	30.50	0.76	0.60
	31.50	0.74	0.62
	32.50	0.71	0.64
***Women***			
	17.50	0.86	0.60
	18.50	0.81	0.65
	19.50	0.76	0.70
	20.50	0.71	0.74
	21.50	0.68	0.77

Abbreviation: GGT = gamma-glutamyl transferase; MetS = metabolic syndrome.

## 4. Discussion

The main result of the present study is that serum GGT can be used as an important predictor of MetS for adults aged 25–65 years living in Beijing. This study mainly evaluated serum liver enzyme levels, including GGT, ALT, AST, and ALP, as risk markers for MetS. Moreover, the optimal cut-off value of GGT for discriminating MetS from normal status was investigated. The majority of similar studies demonstrated the high power of GGT for diagnosing MetS. Moreover, to the best of our knowledge, this is the first study to investigate the optimal GGT cut-off value for the diagnosis of MetS in a population from Mainland China.

As in the findings reported by Hwang *et al*. [21], when samples in the present study were divided into quartiles according to GGT level, high GGT was significantly correlated with a higher prevalence of MetS after adjusting for age, BMI, history of alcoholic fatty liver, and medication use. Similarly, Rantala *et al*. investigated the association between GGT and MetS, reporting a highly significant relationship between GGT and MetS even after adjusting for age, BMI, and alcohol consumption [32]. A 4-year cohort study of 3,698 Korean male workers indicated that elevated GGT could be a sensitive marker of MetS [8]. In another study, serum GGT was associated with the components of MetS [27,33]. Serum GGT is an important predictor of MetS in both men and women without MetS at baseline [34,35]. In a study on Korean adults, the prevalence of MetS, the number of MetS components, and insulin resistance increased as the quartile of serum GGT increased [36].

Modest increase in GGT may be an early marker of cellular oxidative stress via mediation of extracellular glutathione transport into cells of organ systems, or as a mediator of low-grade systemic inflammation and explains the strong association of serum GGT with many cardiometabolic risk factors and diseases [37]. Oxidative stress, assessed by circulating prostaglandin F2α levels, is recognized to be related to obesity [11,38]. The documented predictability of metabolic disorders of MetS, hypertension, and diabetes by GGT activity suggests that, as a reflection of oxidative stress, elevated GGT levels are actively involved in the pathogenesis of these disorders [5].

In the present study, ALT, AST, and ALP were also found to be positively associated with MetS and its components for both men and women. Several emerging studies reported that patients with altered hematological ALT, AST, and ALP status are at high risk of developing MetS and its components [39,40,41]. However, the associations between ALT, AST, ALP, and MetS were weaker than GGT in this study. Also, GGT was more predictive of elevated TG and BMI, compared with the other three MetS components.

According to our results, GGT demonstrates high sensitivity and a partial good specificity for discriminating MetS from non-MetS. New cut-off values of 31.5 and 19.5 U/L GGT were determined for the diagnosis of abnormal MetS conditions in men and women, respectively. Kasapoglu *et al*. reported the optimum cut-off values of 26.5 and 20.5 U/L GGT for diagnosing MetS in a population of Turkish men and women, respectively [42]. Gholamreza derived optimum cut-off values of 20.5 and 16.5 U/L GGT for men and women, respectively, in an Iranian population [29]. The Fifth Korea National Health and Nutrition Examination Survey reported a lower cut-off value of 14 U/L in a Korea population [22], but approximately similar sensitivity and specificity values for the discriminative power of GGT (77.0% and 62.0%, respectively).

There are two limitations to the present study. The first limitation is the lack of WC measurements as an indicator for central obesity. However, BMI was used as a substitute [31], and it has been shown that the two measures of BMI and WC are closely correlated [43]. Another study has indicated that most individuals with an abnormal BMI also have an abnormal WC. Both indices of excess adiposity are positively associated with SBP, FPG, and TG, and inversely associated with HDL-C [44]. Secondly, information about alcohol consumption was not available, and the multivariate model was adjusted for history of alcoholic fatty liver. However, these lifestyle variables will be included in further studies.

## 5. Conclusions

The study confirms the association between GGT and clustered components of MetS. Moreover, our study highlights that, among the four plasma markers of liver injury that were evaluated, GGT is the most strongly associated with MetS in this adult population in Beijing. Moreover, the study indicates that GGT is a sensitive but moderately specific marker for the early diagnosis of MetS in adults living in Beijing. GGT is therefore recommended as a useful diagnostic marker of MetS, because the test is inexpensive, highly sensitive, and frequently measured in clinical practice.
